# Translating Nanotechnology from Bench to Pharmaceutical Market: Barriers, Success, and Promises

**DOI:** 10.1155/2012/678910

**Published:** 2012-05-30

**Authors:** Abhijit A. Date, Rajesh R. Patil, Riccardo Panicucci, Eliana B. Souto, Robert W. Lee

**Affiliations:** ^1^School of Pharmacy and Health Professions, Creighton University, Omaha, NE 68178, USA; ^2^Semler Research Center, Bangalore 560078, India; ^3^Novartis Institutes for BioMedical Research, Cambridge, MA 02139, USA; ^4^Faculty of Health Sciences, Fernando Pessoa University, Rua Carlos da Maia 296, 4200-150 Porto, Portugal; ^5^Centre of Genomics and Biotechnology, Institute of Biotechnology and Bioengineering, P.O. Box 1013, 5000-801 Vila-Real, Portugal; ^6^Pharmaceutical Development and Quality, Particle Sciences, Inc., 3894 Courtney Street, Suite 180, Bethlehem, PA 18017, USA

Nanotechnology is a buzzword of this millennium and it has transformed the face of research in science and technology. The advent of nanotechnology has also influenced the biomedical and pharmaceutical research since last decade. Various nano-architectures have been designed for improving the therapeutic performance of drugs, proteins, peptides, and genes and to achieve their targeting at the site of action. Although nanotechnology has demonstrated dramatic potential in drug delivery research, like any technology, its real success depends on the ability of drug delivery scientists to translate and scale innovations to the commercial pharmaceutical products. It is indeed a very challenging task to successfully overcome manufacturing, clinical, and regulatory hurdles associated with a nanotech product. Nevertheless, the pharmaceutical industry has witnessed commercialization of the nanotechnology-based products for various applications. In the present special issue, we have tried to consolidate various aspects of existing and upcoming nanotechnologies for drug delivery. 

Contribution by V. Morigi et al. takes an overview of business potential and market trend of pharmaceutical nanotechnology. The authors have also discussed financial aspects of nanotechnology by citing noteworthy examples of few nanotech products that have already been commercialized. This contribution could be useful to scientists aiming to start up nanotechnological business ventures. Contribution by N. Anton et al. demonstrates how nanotechnology can change the face of conventional drug delivery systems. In this interesting investigation, the authors demonstrate that coating of conventional tablets with lipid nanoemulsion can be used to modulate the release of the drug from tablet matrix. The paper by P. Severino et al. gives an account of potential of solid lipid nanocarriers for the oral delivery of drugs and peptides. The authors have provided information about the lipids that can be used for oral delivery, role of lipids in the oral delivery, toxicological aspects of lipid nanocarriers, and products under clinical development.

S. Banerjee et al. have given a complete overview of polyethylene-glycol- (PEG-) based conjugates for drug delivery. The contribution fosters understanding design aspects of and chemistry behind PEG-based nano-architectures for drug delivery. Furthermore, the paper has a detailed discussion on the various PEG-conjugates available in the pharmaceutical market. Contribution by A. Garcia et al. highlights the potential of particle replication in nonwetting templates (PRINT), a platform technology based on lithographic techniques for drug delivery applications. The contribution clearly demonstrates potential of PRINT technology to generate particles of various, but precise, morphology for a variety of drugs and biotechnology-based therapeutics (proteins and siRNA). The application of PRINT technology for generating aerosols for pulmonary applications has also been described. This contribution is an example of the attributes required from a nanofabrication technique to circumvent manufacturing-related issues in pharmaceutical nanotechnology.

The paper by F. Lallemand et al. delineates various aspects of and challenges in ocular drug delivery and systematically describes development of Novasorb, a cationic nanoemulsion-based platform ocular delivery system. The authors have furnished a detailed description of the formulation development aspects and ocular safety of excipients which is followed by *in vivo* proof-of-concept and clinical development. This paper gives clear insight into various challenges faced for developing nanomedicine for ocular delivery. The paper by J. D. Heidel and T. Schleup throws light on the various applications of self-assembled nanocarriers consisting of cyclodextrin based polymers. The authors describe various developmental aspects of two platforms based on cyclodextrin-based polymers (Cyclosert and RONDEL) which can enable efficient delivery of drugs or nucleic-acid-based therapeutics. The translational aspects of both the nanocarriers and *in vivo* proof-of-concept have also been furnished in this paper. The research paper by J. Rios-Doria et al. gives insight into developmental aspects of a pH sensitive cross-linked polymeric micelle technology (IVECT). The authors describe synthesis of the polymeric micelles, their ability to encapsulate various drugs, and *in vivo* proof-of-concept for anticancer drugs like daunorubicin and BB4007431.

In a nutshell, we believe that this special issue would give readers insight into various aspects involved in translating nanotechnology from bench to pharmaceutical market. Moreover, the special issue also includes some contributions about nanotechnologies that are currently under clinical development.

*Abhijit A. Date* *Abhijit A. Date* *Rajesh R. Patil* *Rajesh R. Patil* *Riccardo Panicucci* *Riccardo Panicucci* *Eliana B. Souto* *Eliana B. Souto* *Robert W. Lee* *Robert W. Lee*

